# RNA modification: a promising code to unravel the puzzle of autoimmune diseases and CD4^+^ T cell differentiation

**DOI:** 10.3389/fimmu.2025.1563150

**Published:** 2025-03-24

**Authors:** Hui Yu, Zhanchuan Ma, Sensen Su, Zheng Xu, Huanfa Yi

**Affiliations:** ^1^ Central Laboratory, The First Hospital of Jilin University, Changchun, Jilin, China; ^2^ Key Laboratory of Organ Regeneration and Transplantation, Ministry of Education, Changchun, Jilin, China; ^3^ Department of Cardiology, The First Hospital of Jilin University, Changchun, Jilin, China

**Keywords:** post-transcriptional modification, RNA modification, autoimmune diseases, CD4+ T cells, T cell differentiation

## Abstract

Dynamic changes in various forms of RNA modification are critical to the functional homeostasis of the immune system and the pathophysiology of autoimmune diseases. RNA modification-related proteins play an essential role in these processes. At present, the research methods of RNA modification in autoimmune diseases are mainly to detect the expression changes of RNA modification-related proteins in tissues or cells, but there is a lack of explorations of target RNAs and in-depth mechanisms. Considering the important role of CD4^+^ T cell dysfunction in the pathogenesis and progression of autoimmune diseases, the regulatory effect of abnormal RNA modification on CD4^+^ T cells deserves attention, which will provide a perspective for further exploring the mechanism of RNA modification in autoimmune diseases. In this Review, we discuss the abnormal RNA modification changes in patients with autoimmune diseases and highlight the effects of these abnormal changes on CD4^+^ T cells.

## Highlights

- The RNA modification related enzymes may play opposite roles in different autoimmune diseases.
- RNA modification related enzymes exhibit distinct functions across various tissues and cells in autoimmune diseases.
- RNA modification related enzymes play a crucial role in the differentiation of CD4^+^ T cells.
- Targeting RNA modifications may be an emerging approach for treatment of autoimmune diseases.

## 1 Introduction

RNA modifications refer to the chemical labeling of bases or ribose in RNA molecules, involving a selective addition of methyl groups (1). The advancements in transcriptome technology have revealed over 170 chemical modifications in RNA molecules, with methylation being the most predominant type (2). The process of RNA methylation takes place in various kinds of coding and non-coding RNAs, including transfer RNA (tRNA), ribosomal RNA (rRNA), small nuclear RNA (snRNA) and long non-coding RNA (lncRNA). Common modifications found in RNA include N6-methyladenosine (m^6^A), 2’-O-methylation (Nm), and N6-2’-O-dimethyladenosine (m^6^Am). Additionally, there are N1-methyladenosine (m^1^A), 5-methylcytidine (m^5^C) and N7-methylguanosine (m^7^G) (3). The cellular functions of RNA, such as splicing, stability, and translation, are regulated by RNA methylations through their respective regulators known as “writers” (methyltransferases), “erasers” (demethylases), and “readers” (methylation recognition proteins) (4). The characteristics of the major types of RNA modification related proteins have been summarized (**Supplementary Table 1**). Abnormal RNA modifications are closely related to the occurrence and development of many diseases, including cancer, arteriosclerosis, and autoimmune diseases (5). Currently, many scholars focus on the diverse range of biological processes, yet the understanding about the regulatory role of RNA modifications in immune system remains limited.

CD4^+^ T cells are a prominent subset of T lymphocytes, characterized by their abundant presence and diverse functional states (6). They primarily regulate and activate immune responses through antigen recognition and cytokine secretion. The pivotal roles of CD4^+^ T cells in maintaining immune system homeostasis are valued by their associations with various diseases, including inflammatory and autoimmune diseases, such as systemic lupus erythematosus (SLE), rheumatoid arthritis (RA) and inflammatory bowel disease (IBD). The CD4^+^ T cells regulate the occurrence and development of autoimmune diseases through various mechanisms, such as assisting cytokine production and regulating immune response and memory (7). It is of great significance to deeply understand the function and regulatory mechanism of CD4^+^ T cells for further exploring the pathophysiological process of autoimmune diseases and developing new methods of diagnosis and treatment. However, the understanding of which molecular biological processes can mediate the function of CD4^+^ T cells is far more than that, among which the role of RNA modification on their homeostasis and differentiations cannot be ignored.

**Figure 1:**
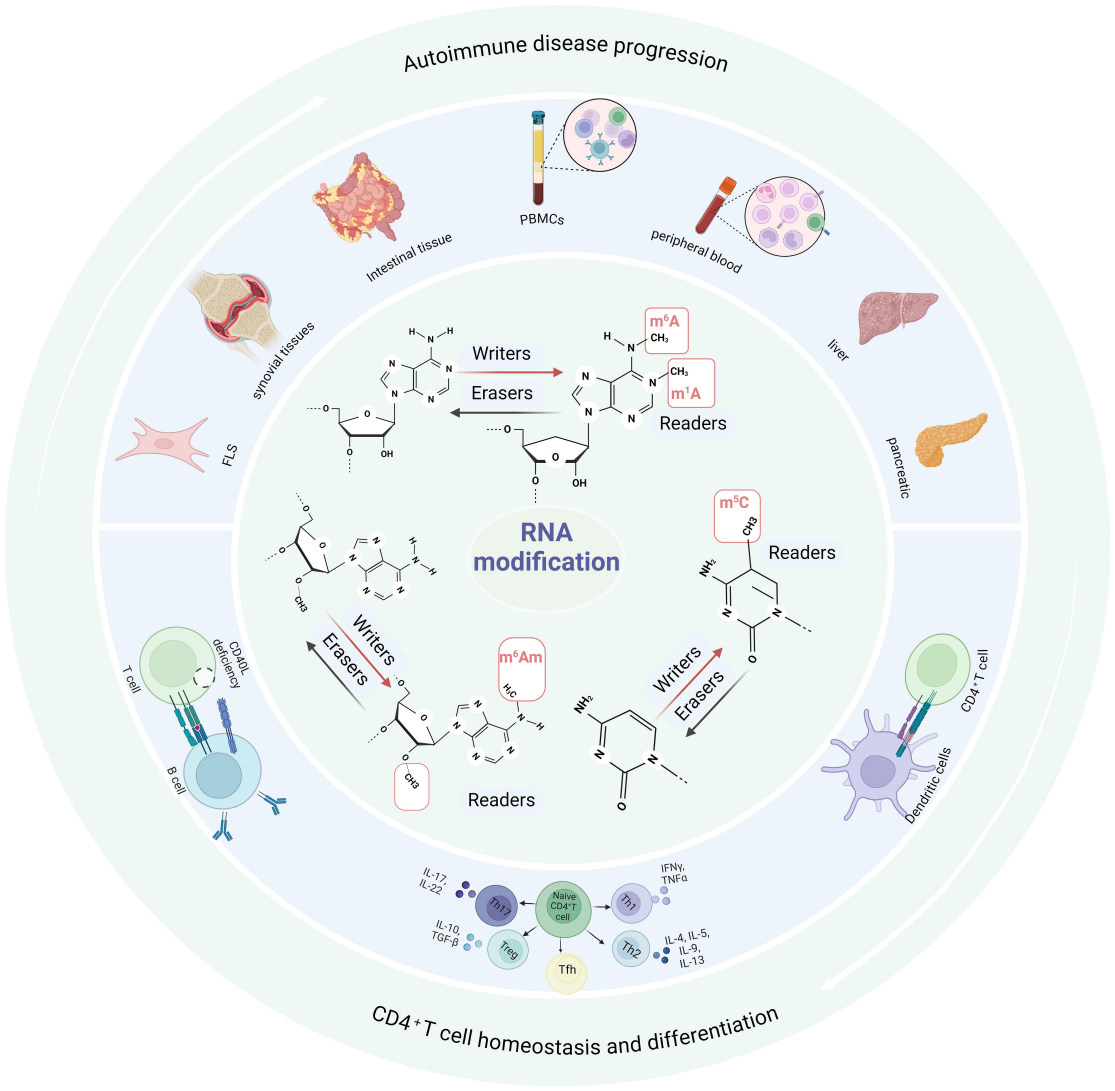
RNA modifications interact with autoimmune disease progression and CD4^+^ T cell homeostasis and differentiation. RNA modification is mediated by writers, erasers and readers. Abnormal RNA modifications are found in peripheral blood, PBMCs, focal tissues and cells in patients with autoimmune diseases or animal disease models. These changes in RNA modification may lead to abnormal CD4^+^ T cell homeostasis and function, which may aggravate the progression of autoimmune diseases. m^6^A, N6-methyladenosine; m^6^Am, N6-2’-O-dimethyladenosine; m^1^A, N1-methyladenosine; m^5^C, 5-methylcytidine; PBMCs, peripheral blood mononuclear cells; FLSs, fibroblast-like synovial cells; IL-17, interleukin-17; IL-22, interleukin-22; IL-10, interleukin-10; IL-4, interleukin-4; IL-5, interleukin-5; IL-9, interleukin-9; IL-13, interleukin-13; TGF-β, transforming growth factor-beta; TNF-α, tumor necrosis factor-alpha; IFN-γ, interferon gamma; Th, helper T cells; Tfh, T follicular helper cells; Tregs, regulatory T cells.

In this Review, we explore the abnormal RNA modification changes in patients with autoimmune diseases and highlight the effects of these changes on CD4^+^ T cells (**Figure 1**). In addition, we discuss the regulatory effects about RNA modification-related proteins on CD4^+^ T cell differentiation, and point out the key points worthy of further study in the future.

## 2 Important types of RNA modifications

### 2.1 m^6^A

The m^6^A modification occurs at the 6^th^ position of the adenine ring in RNA as the most prevalent and abundant post-transcriptional modification in eukaryotic mRNA (1). The m^6^A modification is predominantly localized within the 3’-untranslated region (3’-UTR) proximal to the coding sequence (CDS) and stops the codon of mRNA, highlighting the crucial role in mRNA biology (8, 9).

The m^6^A “writers” complex mainly comprises three core proteins: methyltransferase-like 3 (METTL3), METTL14, and Wilms’ tumor-associated protein (WTAP) (2). METTL14 and METTL3 form a heterodimer, and METTL14 is primarily responsible for recognizing and localizing subunits (10). The WTAP facilitates m^6^A modification by recruiting co-localized METTL3-METTL14 heterodimers within nucleolar patches. Other “writers” complex include METTL5, METTL16, RNA binding motif protein 15/15B (RBM15/15B), vir-like m^6^A methyltransferase associated (VIRMA), and Zinc finger CCCH-type containing 13 (ZC3H13) (11), which are crucial for the nuclear localization and stabilization.

The two “erasers” include the Fat mass and obesity-associated protein (FTO) and AlkB homologue 5 (ALKBH5), which can mediate reversible demethylation (12). The three “readers” groups include the YTH-RNA binding domain family (YTHDF), consisting of YTHDF1/2/3 and YTHDC1/2, the heterogeneous nuclear ribonucleoprotein (HNRNP) family and insulin-like growth factor-2 mRNA-binding proteins family (IGF2BPs), consisting of IGF2BP1/2/3 (13).The interactions between YTHDF3 and YTHDF1 enhance RNA translation, while the binding of YTHDF3 to YTHDF2 promotes RNA degradation (14). YTHDF1 fosters the translation of mRNA while YTHDF2 encourages the degradation of mRNA, but the mechanism remains unclear (14). IGF2BP2 stabilizes the mRNAs containing m^6^A and facilitates their translation (15).

### 2.2 m^6^Am

The m^6^Am is an RNA modification located immediately after the m^7^G cap structure of mRNA, specifically on the first nucleotide (16).

The m^6^Am “writers” complex comprises phosphorylated CTD-interacting factor 1 (PCIF7) and METTL4. It’s reported that the m^6^A demethylase FTO could also demethylate the m^6^Am (17). The m^6^Am differs from m^6^A solely in the methylation on the 2’-O position of the ribose. Despite this distinction, the overall structures of m^6^Am and m^6^A are highly similar. (17). Due to the similar chemical structure of m^6^Am and m^6^A, m^6^A-specific antibodies often recognize m^6^Am as well (16). The localizations of FTO are dependent on the cell cycle stages and regulated by Casein kinase II-mediated phosphorylation. Since the higher m^6^A levels in cells than m^6^Am, the effects of FTO are attributed to m^6^A activity (18).

### 2.3 m^1^A

The m^1^A researches have revealed predominant distributions in tRNAs and rRNAs (19). The methyltransferases, known as “writers”, include TRMT6, TRMT61A/B, TRMT10C, and NML (20). The demethylases, referred to as “erasers”, include ALKBH1/3/7 and FTO, and the “readers” include YTHDF1/2/3 and YTHDC1 (21).

The TRMT6/61A complex, located in the cytoplasm, can methylate cellular tRNA m^1^A58 (T-loop) and mRNA with T-loop-like structure (20). The NML, localized in the nucleus, can methylate m^1^A on 28S rRNA (22). ALKBH1 demethylates most m^1^A in cyto-tRNAs with the m^1^A58 in tRNAs as a significant substrate (23).YTHDF1/2/3 and YTHDC1 can recognize the common modification m^6^A (20). However, their binding affinity to m^1^A is weaker than that of m^6^A. Researches on their function specifically as “readers” of m^1^A are still limited.

### 2.4 m^5^C

The m^5^C refers to the process by which methyltransferase uses SAM as a methyl donor and transfers methyl groups to cytosine. There are known m^5^C methyltransferases, including the NOL1/NOP2/sun domain (Nsun) family, DNA methyltransferase (DNMT) homologs and tRNA-specific methyltransferase (TRDMT) families (24). The majority of tRNAs are methylated by Nsun2 at the variable loop, and specifically in leucine at the wobble position (24).

To date, the role of m^5^C “erasers” remains a subject of controversy. Enzymes belonging to the ten-eleven translocator family (TET) have been reported to oxidize m^5^C in mRNA (25), resulting in the production of 5-hydroxymethylcytosine (hm5C) (25). Additionally, ALKBH1 catalyzes the formation of oxidation of 5-formylcytosine (f5C) at the mitochondrial tRNA wobble position (24). Although the f5C depositions in mitochondrial tRNAs are well-established biologically, the biological relevance in terms of hm5C deposition in mRNAs remains unclear (26). Aly/REF export factor (ALYREF) and Y-box-binding protein 1 (YBX1) appear to be m^5^C “reader” proteins that govern mRNA fate based on its m^5^C status (27).

### 2.5 m^7^G

According to locations, there are two types of m^7^G modification. The one type is m^7^G-cap at 5’-terminal and the other is internal m^7^G modification (28). METTL1, WD repeat domain 4 (WDR4), RNMT and RAM as “writers” can catalyze m^7^G modification (29). However, other enzymes still need to be identified in the future.

### 2.6 Nm

The known 2’-O-methylation (Nm, where N represents any nucleotide) involves the addition of a methyl group (-CH_3_) to the 2’-hydroxyl (-OH) of the ribose moiety. This modification is highly conserved and abundant, occurring at multiple sites in tRNA, rRNA, and snRNA (30).

The Nm “writers” FtsJ RNA 2’-O-methyltransferase 1 (FTSJ1) exhibits high expression levels in tRNAs, while fibrillarin shows elevated expression levels in rRNAs, both of which have significant promoting effects on cellular growth. The Nm “erasers” and “readers” were unknown (31). The enrichments of Nm in rRNAs confer protection against hydrolysis and enhance the stability of their conformation.

## 3 RNA modification-associated enzymes: regulate autoimmune diseases pathophysiological process

RNA modifications encompass various stages of RNA metabolism, such as RNA splicing, transport, editing, transcription, translation and degradation. Currently, primary emphasis in the field of RNA modifications lies within tumor studies, while autoimmune diseases have received comparatively less attention. Then, we summarize the role of RNA modification in autoimmune diseases and explore possible therapeutic ideas.

### 3.1 Systemic lupus erythematosus

SLE is a complex autoimmune disease involving multiple systems, characterized by the production of numerous autoantibodies, loss of tolerance, and tissue damage (32). The involvement of m^6^A modification-related proteins in the regulation of SLE disease progression is multifaceted. The mRNA levels of *METTL3*, *WTAP*, *ALKBH5*, *FTO* and *YTHDF2* in peripheral blood of SLE patients are significantly lower than healthy controls, while *METTL14* is unchanged (33). The decreased mRNA level of *ALKBH5* in peripheral blood is a risk factor of SLE, which are associated with anti-dsDNA and anti-nucleosome, rash (33). However, these changes are not exactly the same in peripheral blood mononuclear cells (PBMCs). The mRNA expression of *METTL14*, *ALKBH5*, and *YTHDF2* in PBMCs of SLE patients is decreased, while others are unchanged (34). The decreased level of *YTHDF2* in PBMCs is a risk factor for disease progression (34). Although the causes are complex, the reason for these differences can lie in neutrophils. Neutrophils are crucial players in SLE pathogenesis, high numbers of immature neutrophils and dysregulated neutrophil death present in the blood of SLE patients (35). The activation of neutrophils can be regulated by METTL3-mediated m^6^A modification (36). Although the underlying mechanism remains unclear, it is likely that the specific transcriptome locations and downstream targets play a pivotal role in determining its functionality.

### 3.2 Rheumatoid arthritis

RA is an autoimmune disease characterized by symmetrical inflammations in bilateral joints, leading to persistent pain and causing damage to bone and cartilage tissues (37). Researchers evaluate the levels of whole-transcriptome m^6^A modification in the synovium from RA patients and healthy controls, and find that the m^6^A is closely related to RA inflammation (37). The m^6^A methylation level is increased in synovial tissues and fibroblast-like synovial cells (FLSs) from RA patients (38).

In comparison to normal controls, the level of *METTL3* mRNA is up-regulated in PBMCs, macrophages, synovial tissues, and FLSs from patients with RA (38–40) Elevated *METTL3* mRNA level in PBMCs may indicate high disease activity (39). METTL3 promotes the tumor-like growth of RA-FLSs (38). The proliferation and invasion of *METTL3*-deficient FLSs are significantly attenuated (40). The NF-κB signaling pathway exerts a crucial influence on these effects through the regulation of the secretion of inflammatory mediators (39, 41). Some drugs like Artemisitene can inhibit RA progression by targeting METTL3-mediated m^6^A modification (42, 43).

Surprisingly, METTL14 seems to have the opposite effect to METTL3 in PBMCs. The mRNA level of *METTL14* is decreased in PBMCs of RA, and the inhibitory factors of NF-κB inflammatory pathway are methylated (44). Although it is difficult to explain the mechanism, the varying stages of disease progression in patients may constitute a contributing factor. Similarly, the opposite effects of METTL3 and METTL14 are also present in the course of hepatocellular carcinoma (45). The expression of METTL14 is up-regulated in macrophages, synovial tissues, and FLSs from patients with RA (38, 46, 47). These may be related to *METTL14* mRNA enhancing the migration and invasion ability of FLSs and promoting the M1 polarization of macrophage (46, 47). The mRNA level of *METTL14* in peripheral blood is increased (46) or without significant change (48), which may be due to the different stages of disease progression in patients. WTAP is up-regulated in synovial tissue and FLSs from patients with RA (38). WTAP promotes FLSs pyroptosis and related inflammatory responses via NLRP3 (49). However, the peripheral blood mRNA level of *METTL3*, *METTL14*, and *WTAP* reveals no significant differences from RA patients and healthy individuals (48). The causes are complex, potentially due to the association with neutrophils. Neutrophil derived reactive oxygen species (ROS) and granuloprotease cause cartilage injury in RA and are associated with pathogenic post-translational modification (35). METTL3, METTL14 and WTAP may be expressed in neutrophils and play a complex role in regulating the disease progression of RA.

ALKBH5 and FTO are upregulated in FLSs and synovial tissue from RA patients (50, 51). The *JARID2*, one of the targeting mRNA of ALKBH5-mediated m^6^A modification, can negatively regulate the migration, invasion and proliferation of FLSs (50). FTO promotes *ADAMTS15* mRNA stability in an m^6^A-IGF2BP1 dependent manner, which negatively regulates the inflammatory response, migration and invasion of FLSs (51). The upregulation of ALKBH5 in FLSs can enhance the proliferation, migration, and inflammatory response (52), while these capabilities are completely reversed in the absence of FTO (51). However, the mRNA level of *ALKBH5* and *FTO* is significant downregulated in peripheral blood from RA patients (48). The changes in ALKBH5 and FTO, whether increasing or decreasing, exhibit a consistent trend, suggesting their potential synergistic role. The aforementioned findings indicate the variations in RNA modification-related proteins across different cells in RA, while the specific methylated genes that play a crucial role remain unknown.

The mRNA level of *YTHDF2* in peripheral blood and PBMCs of RA is significantly decreased (48, 53). The expression of *YTHDF2* mRNA is negatively correlated with multiple inflammatory markers (53). The *NLRP3* is a target gene on ALKBH5/YTHDF2- mediated m^6^A modification, which has been shown to be closely related to RA (54). The level of *IGF2BP3* mRNA is increased in synovial tissue and PBMCs of RA, while after treatment, *IGF2BP3* mRNA level is decreased, which suggests that *IGF2BP3* might be a new potential target during the treatment of RA (55). The mRNA level of *IGF2BP2* is increased in synovial tissue of RA (56).

### 3.3 Inflammatory bowel disease

IBD, consisting of Crohn’s disease (CD) and ulcerative colitis (UC), is a chronic, relapsing inflammatory disorder (57).

The m^6^A-methyltransferases in IBD exhibit similar pro-inflammatory effects. METTL3 is up-regulated in the pathological tissues of IBD patients. The inhibition of *Mettl3* or *Mettl14* in intestinal epithelial cells (IECs) can effectively inhibit NF-κB signaling and significantly relieve inflammation (58, 59), which demonstrates the synergistic effect of *Mettl3* and *Mettl14*. Similarly, the deficiency of *Mettl3* in macrophages can ameliorate dextran sulphate sodium (DSS)-induced colitis in mice (60). Mechanistically, the depletion of *Mettl3* enhances the Ythdf3-mediated PGP expression, resulting in the suppression of Th1 differentiation (60). Moreover, Wtap can promote the disease progression of DSS-induced IBD in mice (61).

The expression of *ALKBH5* mRNA in intestinal mucosa of RA is contradictory and complex, due to the different stages of disease progression, the consistent conclusion could not be reached. The mRNA level of *FTO* is reduced in intestinal mucosa of RA and negatively correlated with Mayo score (62). FTO seems to exhibit mainly anti-inflammatory effects. *Fto* deficiency leads to more severe colitis in IECs under DSS induce (62).

IGF2BP1 and IGF2BP2 are down-regulated in CD or UC tissues compared to normal tissues (63). However, IGF2BP1 and IGF2BP2 appear to play an anti-inflammatory role. The *Igf2bp2*-knockout macrophages exhibit the enhanced M1 phenotype and promote DSS-induced colitis development (64).

### 3.4 Ankylosing spondylitis

The rheumatic chronic inflammatory disease known as Ankylosing spondylitis (AS) is characterized by inflammatory spondylitis, peripheral arthritis, and enthesitis (65). The mRNA expression of *YTHDF2* and *ALKBH5* in PBMCs of newly diagnosed AS patients is significantly decreased, and the expression of *YTHDF*2 mRNA in PBMCs is a risk factor for AS (66). The total m^6^A level and METTL14 expression are decreased in T cells of AS. Mechanistically, the expression of FOXO3a reduces with the decrease of METTL14, leading to increased autophagy and inflammation (67). The mRNA level of *WTAP* in PBMCs of AS patients is up-regulated. *HNRNPC* is down-regulated, which are potential m^6^A regulators of AS (65). However, the RNAs and signaling pathways targeted by these proteins remain unclear.

### 3.5 Primary Sjogren’s syndrome

Primary Sjogren’s syndrome (pSS) is a chronic autoimmune disease of unknown etiology, defined by dry mouth syndrome and dry keratoconjunctivitis (68). The m^6^A, m^1^A, m^5^C, and m^7^G modification play a critical role in the diversity and complexity of the SS immune microenvironment (69). The total m^6^A level and METTL3 expression are increased in PBMCs of SS, and the upregulation of METTL3 is associated with disease severity (68). The elevated *ALKBH5* mRNA in PBMCs of SS patients may be a risk factor (70).The mRNA levels of *METTL3*, *ALKBH5* and *YTHDF1* in T cells of SS patients are significantly up-regulated (71). The mRNA level of *ALKBH5*, *RBMX*, *RBM15B* and *YTHDF1* is down-regulated in peripheral blood and labial salivary gland tissues of SS patients (72). METTL3 and METTL14 are elevated in salivary gland epithelial cells of SS patients, but METTL3 may play an anti-inflammatory role. The inhibition of *METTL3* increased inflammatory related-gene expression, whereas inhibition of *FTO* presents the opposite effect (73). However, there is a lack of *in vitro* experiments to explore the mechanism.

### 3.6 Other autoimmune disease and chapter summary

YTHDF2 is significantly up-regulated in the liver of autoimmune hepatitis (AIH) patients, which is related to the degree of inflammation, and *RXRα* is one of the target RNA for this experiment (74). METTL3 is increased in pancreatic-β cells from patients with type 1 diabetes (T1D), the genes associated with innate immune mediators including *OAS1*, *OAS2*, *OAS3*, and *ADAR1* exhibit hypermethylation (75).

In summary, RNA modification-related enzymes are changed in autoimmune diseases, and the role of RNA modification in autoimmune diseases deserves attention (**Supplementary Table 2**). Current researches have mainly concentrated on alterations in the expression of RNA modification enzymes in cells, tissues, and organs, while neglecting the upstream and downstream mechanism associated with changes in enzyme activity (33, 34, 46, 48). However, further investigations into the underlying mechanism are limited. Firstly, RNA modification-related enzymes may change differently with disease progression (46, 48). The mRNA level of *METTL14* in PBMCs from SLE patients is significantly reduced, whereas it remains unchanged in the overall peripheral blood (33, 34). These two articles are from the same team, and it can be inferred from the above conclusions that the level of *METTL14* mRNA in neutrophils and red blood cells is different in patients and healthy controls. In other words, the extent of RNA modification may vary among different cell types, which should be considered when developing therapeutic strategies targeting RNA modifications for disease treatment in the future. These regulatory proteins such as METTL3, METTL14 and WTAP may act simultaneously or independently, and it is necessary to explore the dominant actors causing the changes. However, the relevant researches are limited at present, which is worthy of further exploration. Additionally, existing studies have primarily focused on investigating alterations in the expression of RNA modification enzymes in cells, tissues, and organs from both patient and healthy control samples. However, the upstream and downstream mechanisms responsible for these changes in enzyme activity remain to be elucidated. Investigating the localization of RNA modifications within the transcriptome can enhance our understanding of how these proteins impact disease progression and enable exploration into upstream factors regulating these enzyme modifications. Further investigations both *in vitro* and *in vivo* are necessary to determine the localization of different RNA modifications within multiple cellular transcriptomes from patients with autoimmune diseases, to identify target RNAs affected by these modifications, and to assess their influence on disease activity as well as drug toxicity and organ damage. Lastly, current researchers focus on m^6^A modification that involves more than twenty regulatory proteins, only METTL3, METTL14, ALKBH5, and FTO have been elucidated thus far. The role of other RNA modification needs further investigation regarding autoimmune diseases.

## 4 The essential role of CD4^+^ T cells in autoimmune disease

The abnormality in immune cell function and composition in human diseases have been a focal point of recent researches, and significant advancements have been made. CD4^+^ T cells, which differentiates into helper T cells (Th), T follicular helper (Tfh) and regulatory T cells (Tregs), play an essential role in autoimmune diseases. The balance of Th1/Th2 and Th17/Treg has attracted a lot of interest over the past decades. Aspects of the imbalance between Th1/Th2 and Th17/Treg in autoimmune disease are summarized in the Reviews (76, 77). We will not discuss the above topics here. Our team is mainly committed to exploring the study of Th17 and myeloid-derived suppressor cells (MDSC) on autoimmune diseases (78). Next, we will mainly review the effect of RNA modification on CD4^+^ T cell function.

Recent research shows that the increased level of Th17 cells in the circulation and tissues of SLE patients is closely associated with the defects of apoptosis (79). Current researches on autoimmune disease involve the Th17 differentiation signaling pathway regulated by methylation. For example, the hypomethylation and hypermethylation of Th17-related genes serve as indicators for the presence of intestinal CD4^+^ T cells in patients with CD (80). SMARCA5, belonging to the SWI/SNF family, can promote the development of UC through the methylation processes (81). The recent findings demonstrate that RNF180 can exacerbate colon inflammation and disrupt the Th17/Treg cell balance with UC by modulating the ALKBH5/SMARCA5 axis (82).

The emerging T cell subsets such as Th9 and Th22 cells, link the inflammatory factors to autoimmune disease. The increased expression of IL-9 in RA is associated with heightened disease severity (83). The transcription factor PU.1 can directly target FMS like-tyrosinekinase-3 (FLT3) in macrophages and FLSs to promote the development of RA (84). In SLE patients, the expansion of CXCL13^+^ Tph/Tfh cells is associated with disease activity, while IL-22^+^ Th22 cells decrease (85). The formation of AhR and JUN in CD4^+^ T cells inhibits the expression of CXCL13 and induces Th22 differentiation (85). However, there is no direct evidence that Th9 and Th22 are regulated by RNA modification.

In the germinal centers (GCs), Tfh regulates B cell maturation triggered by T cells (86). Tfh cells secrete IL-21, which is to be crucial for supporting GCs. The mRNA expression of transcription factor *Bcl-6* is elevated in patients with RA and IBD (87). The defective ubiquitination of CBL and CBLB leads to impaired Bcl-6 function, resulting in the dysregulation of Tfh cells in SLE patients (88). Previous studies indicate that the regulation of Bcl-6 involves histone methylation, acetylation (89) and β-hydroxybutyrylation modification (90).

In conclusion, CD4^+^ T cells are the central “regulators” of the immune system and play an important role in autoimmune diseases. Prior researches have demonstrated that CD4^+^ T cells undergo epigenetic modifications. Although these are preliminary explorations, they establish a foundational understanding of the role of RNA modifications in CD4^+^ T cells. Next, we delve into the role of RNA modification-associated proteins on CD4^+^ T cell homeostasis and differentiation.

## 5 RNA modification-associated enzymes: regulate CD4^+^ T homeostasis and polarizations

Researchers have examined overall changes in RNA modification in patients with autoimmune diseases. The increased levels of Am, m^1^A, m^6^A, and 3 ‘OMeA in CD4^+^ T cells from SLE patients, while decreased levels of m^3^C, m^5^C, m^1^G, m^5^U, and t^6^A compared with healthy controls (91). In this study, the specific roles of various regulatory proteins remain unclear. Next, we will comprehensively examine the impact of these RNA modification-related proteins on CD4^+^ T cells, categorized by the three types.

### 5.1 Writers

Different from previous research (91), the total m^6^A level of CD4^+^ T cells is reduced in SLE patients compared with healthy controls, which is mainly caused by the decreased mRNA level of *METTL3*. The degree of reduced *METTL3* is correlated with disease activity (92). Although these researches are difficult to explain mechanistically, it is possible that distinct subpopulations of CD4^+^ T cells undergo different methylation changes. In other words, the same RNA modification protein may exert diverse effects in different types of CD4^+^ T cells.

Mettl3 seems to exhibit important pro-inflammatory or anti-inflammatory effects in different types of CD4^+^ T cells. The proportion of Th2 cells in *Mettl3*-knockout CD4^+^ T cells is increased, while the Th17 is decreased (93). The *Mettl3*-knockout naïve T cells lose the ability to induce colitis phenotypes (93). The mechanisms are involved in the elevated levels of Janus kinase (JAK)-STAT5 signaling pathway related genes, such as *SOCS1*, *SOCS3*, and *CISH*. These genes belong to the *SOCS* family, targeted by m^6^A modification to prolong half-life and enhance gene stability, can negatively regular the T cell-related signaling pathway (94, 95). After inducing differentiation of different effector T cells *in vitro*, the expression of Mettl3 is increased dramatically in the Tregs (92). The inhibition of Mettl3 reduces *Foxp3* mRNA level by decreasing its m^6^A modification and mRNA stability, suppressing Tregs differentiation. In addition, the vivo experiments confirmed that Mettl3 deletion increased antibody production and worsened the lupus-like phenotype in chronic graft versus host disease (cGVHD) mice (92). Importantly, the deficiency of *Mettl3* in Tregs results in the elevated levels of *Socs* family genes and suppresses the biological activity of Tregs by inhibiting IL-2/Stat5 signaling (96). Mettl3 plays a key role in the formation of GCs by activating the endogenous pathway of Tfh programming and inhibiting Th1 lineage related genes. The deficiency of *Mettl3* in CD4^+^ T cells impairs the differentiation of Tfh cells and the formation of GCs. Mechanistically, the *Mettl3* enhances the stability of *Tcf7* mRNA through catalyzed m^6^A methylation at 3’ UTR, improving the expression and function of TCF-1 to promote Tfh differentiation (97). In humoral immunized mice, the pharmacologically inhibiting of Mettl3 decreases the proportion of naïve T cells while increases the proportion of effector T cells. To be specially, the frequencies of Tfh cells and Treg cells in spleen are significantly reduced, which may be related to the decreased expression and stability of *Foxp3* (92).

Mettl14 seems to exhibit mainly anti-inflammatory effects. The mice with the *Mettl14*-knockout T cells exhibit an increased proportion of Th1 and Th17, and the impaired function of Tregs. The expression of RORγt is reduced in *Mettl14*-knockout Tregs. In addition, the *Mettl14*-knockout naïve T cells lose their potential for induction into Treg cells (98, 99). Mechanistically, these changes are associated with activation of the mTOR pathway, a signaling pathway known to inhibit Tregs function (99). Moreover, in experimental transplantation mouse models (EAEs), the down-regulated expression of METTL14 facilitates the infiltration of CD4^+^ T cells surrounding the allograft, inducing accelerated rejection (100).

The *Wtap*-knockout T cells result in colitis phenotypes more severe than wild types, and the *Wtap*-knockout Tregs obtaine increased expression of Rorc and Il-17a, which is involved in the TCR signal activation and conduction (101). These phenotypes following *Wtap* deletion are reproduced on *Virma* or *Mettl3*-knockout models, suggesting that these phenotypes result from the changes in m^6^A signaling rather than non-m^6^A dependent effects of Wtap (101). At the same time, the non-m^6^A dependent effects of WTAP are also remarkable. For example, WTAP is upregulated in CD4^+^ T cells of tolerant kidney transplant recipients and positively correlated with Tregs proportion (102). However, crucial inquiries regarding the precise localization of WTAP-mediated m^6^A modification within the T cell transcriptome and the specific target genes influenced by m^6^A remain unresolved.

Among tRNA modifications, some studies have shown that the m^1^A58 modification of tRNA (tRNA-m^1^A58) can improve the initiation and elongation efficiency of protein translation (103, 104). The tRNA-m^1^A58 modification level is much higher than other tRNA methylation in activated T cells (105). Trmt61a and Trmt6 seem to exhibit important pro-inflammatory effects in different types of CD4^+^ T cells. Trmt61a and Trmt6 are up-regulated during T cells activation, which enable m^1^A RNA modification on specific early expression tRNAs subsets. The *Trmt61*-knockout mice could not be induced to develop a colitis phenotype, resulting in a predominant arrest of CD4^+^ T cells in the G_0_-G_1_ phase (105). However, the key questions regarding the precise localization of Trmt61a and Trmt6-mediated m^1^A modifications in the T cell transcriptome remain unresolved.

It has been reported that RNA m^5^C methylation has a potential regulatory effect on homeostasis regulation of Th17 cells (91, 106). RORγt facilitates the formation of transcription-coupled m^5^C modification on Th17 cell-specific cytokine mRNA by recruiting Nsun2 to the chromatin region of the target gene. Furthermore, the *Nsun2*-knockout T cells diminish the secretion of proinflammatory cytokines and impair the intercellular communication between Th17 cells and IL-17 receptor-expressing cells, impeding colitis progression (107). At present, the researches of m^5^C effect on CD4^+^ T cell proliferation and differentiation are limited, whether m^5^C “writers” independently or jointly affect T cell function are still worthy further study. It is imperative to further investigate the location of m^5^C modifications in the T cell transcriptomes, whether these modifications affect thymocytes development, and whether they affect IL-7 or TCR signaling in mature T cells.

### 5.2 Erasers

Although, there are no significant changes in the mRNA levels of *Fto* among effector CD4^+^ T cells compared to the naïve CD4^+^ T (108), the involvement of FTO in CD4^+^ T cell homeostasis and differentiation should not be dismissed. Importantly, the m^6^A plays a crucial role in the regulation of the expression of CD40L in CD4^+^ T cell, and FTO directly influences the expression of CD40L (12). The primary role of CD40L is to bind with CD40 on the APCs, initiating the activation of the immune response (109). Currently, there are ongoing studies investigating the development of inhibitors targeting CD40L in SLE, RA, and other autoimmune diseases (110). In addition, the *Fto*-knockout IECs under the DSS-induced results in heightened severity of colitis, with a higher proportion of Th17 cells than in wild types (62). These findings suggest that FTO may indirectly regulate the function of CD4^+^ T cells. Similarly, there is an up-regulation of FTO in patients with aortic dissection (AD), which is inversely correlated with the abundance of activated CD4^+^ T cells (111). However, the mechanisms require further investigation.

It is worth noting that studies have demonstrated that FTO ubiquitously expresses in various developmental stages of human tissues, localizing to both the cytoplasm and nucleus (112, 113). The expression is highest in the hypothalamus of human brain tissue, suggesting the important role for FTO protein in metabolic control (114). Thus, the alterations mentioned above may arise from the combined influences of metabolic adjustments and RNA modification. FTO protein is initially identified as a regulator of lipid metabolism (115). The disorders of lipid metabolism have been reported in autoimmune diseases (116, 117). *Fto*-knockdown in IECs leads to upregulated m^6^A level of *CerS6 (Ceramide synthase 6)* and decreased C_16_-ceramide synthesis, accelerating pro-inflammatory macrophage and Th17 differentiation and promoting inflammation (62). In this study, FTO plays a role in regulating sphingolipid metabolism and m^6^A modification. On the one hand, it is needful to explore whether FTO-mediated methylation at specific loci causally impacts lipid metabolism and whether these changes are critical for T cell homeostasis. In the future, there is a need to development more precise methods to distinguish the sites between of FTO-mediated methylation and lipid metabolism. On the other hand, considering that FTO is not only involved in the regulation of m^6^A, but also serves as a crucial demethylase for m^6^Am, m^1^A and m^5^C modification (118, 119). It is crucial to distinguish the sites of these RNA modifications mediated by FTO.

ALKBH5 can decrease the m^6^A modification in *CXCL2* and *IFN-γ* mRNA, leading to enhanced transcriptional stability and protein expression. Consequently, the deficiency of Alkbh5 in EAEs results in the impairment of the IL-17 signaling pathway in CD4^+^ T cells, and the augmented CD4^+^ T cell response increases the infiltration of neutrophils into the central nervous system during neuro-inflammation (108). The impacts of Alkbh5 and Mettl3 on CD4^+^ T cell-mediated autoimmunity models exhibit a similar pattern, yet the underlying mechanism remains incompletely understood, the possible reason could be attributed to the fine-tuning of T cell activity through a temporal-sequential ordering by these two molecules. Whether ALKBH5 and FTO participate in these processes synergistically, and whether the changes caused by *ALKBH5*-knockout can be reversed through overexpression of *FTO*, are also worth further investigation. Although ALKBH1, ALKBH 3 and ALKBH5 belong to the AlkB homologue family, the role of ALKBH1and ALKBH3 in the development, and function of CD4^+^ T cells remains unexplored. The self-regulation processes of CD4^+^ T cells are highly intricated, exploring the role of “erasers” in this series of processes will hold significant importance in bridging the gaps within related fields.

### 5.3 Readers

Most of the function and mechanism of RNA methyltransferase and demethylase are mediated by methylation recognition proteins. In tumor immunity, these proteins are considered potential therapeutic targets. Therefore, it is meaningful to explore the functions of “readers”. So far, no studies have directly investigated the effect of YTH family proteins on CD4^+^ T cell homeostasis and differentiation by knocking down *YTH family genes* in CD4^+^ T cells, and the existing studies can only provide indirect evidence. YTHDF1/2/3 proteins in naïve CD4^+^ T cells recognize m^6^A-modified *SOCS family genes* and promptly initiate the degradation of target genes (120). Subsequently, the expression changes of SOCS further affect the JAK/STAT signaling pathway and regulate T cell homeostasis. DCs are the main APCs responsible for T cell activation (121). Silencing *Ythdf1* in DCs can impact T cell activation by diminishing the expression of co-stimulatory molecules CD40 and CD80, however, these changes don’t occur in *Mettl3*-knockout DC cells (122). Ythdf1 can recognize m^6^A-modified mRNAs and promote the mRNA translation of *CD40* and *CD80*. In some cases, dysfunction in m^6^A readers may not cause changes in overall m^6^A modification levels, the function of “readers” should be better understood by considering “writers” and “erasers” together. Considering the crucial role of “readers” in the RNA modification system and the contributions to precise regulation of immune response, it is imperative to further investigate their underlying mechanisms.

## 6 Conclusions and future perspectives

**Figure 2:**
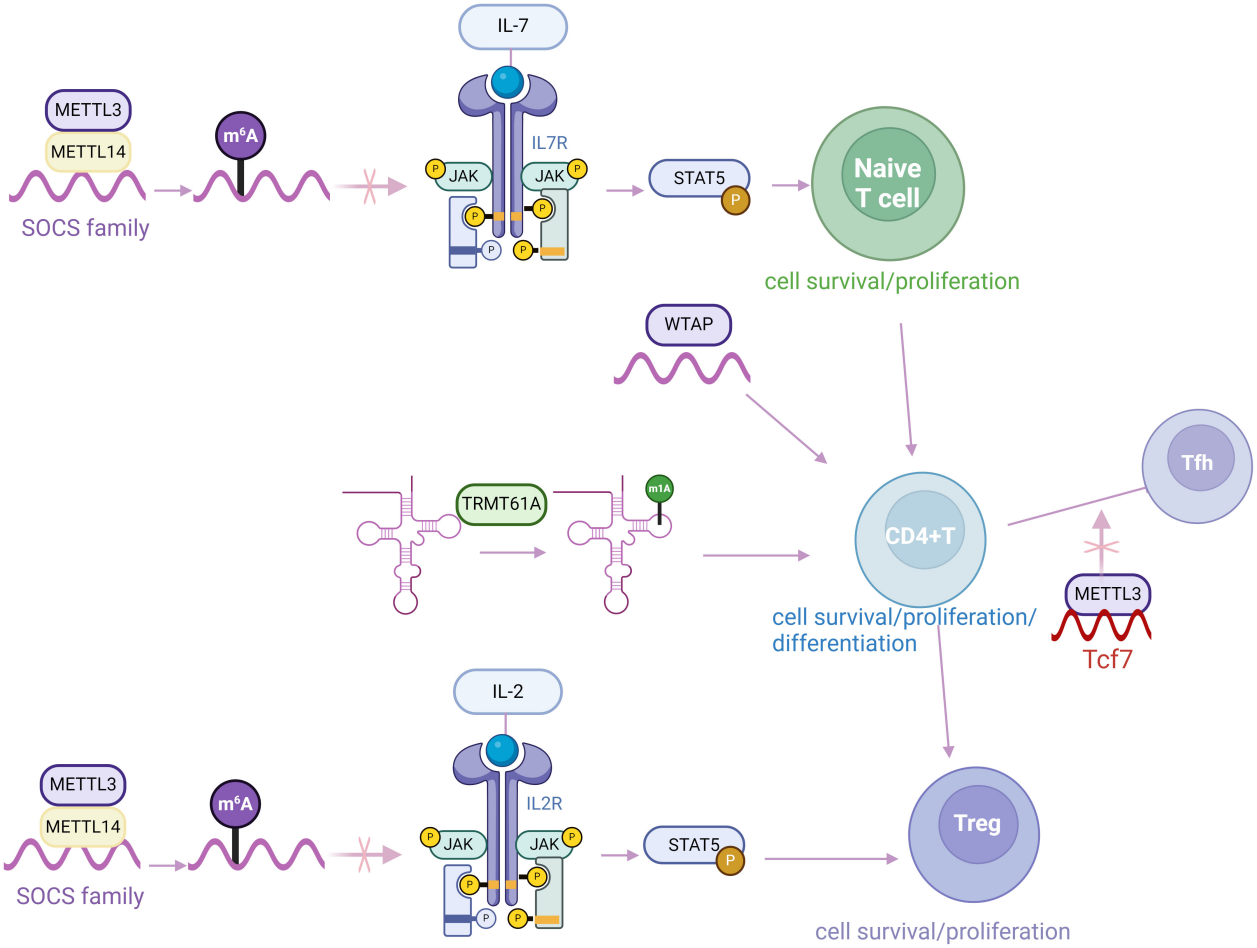
RNA modification plays a crucial role in regulating the development of CD4^+^ T cell. m^6^A, N6-methyladenosine; m^1^A, N1-methyladenosine.

To conclusion, despite the variety and complexity of RNA modification associated proteins, m^6^A-associated proteins are currently the most studied than others (123). The components of m^6^A modification play a crucial role in autoimmune responses and the differentiation of CD4^+^ T cells, which offers potential novel insights into the treatment of various diseases by regulating physiological and pathological processes associated with CD4^+^ T cells (**Figure 2**). Previous studies primarily utilize a conditional knockout mouse model targeting specific genes in CD4^+^ T cells to explore their impacts on cell activation, proliferation, and differentiation. However, their pro-inflammatory or anti-inflammatory effects also depend on the specific target RNAs and the type of autoimmune disease.

Limited research has been conducted on other types of RNA modification, highlighting the need for further investigation into the role of m^1^A and m^5^C modified protein in controlling CD4^+^ T cell function and autoimmune disease. Nevertheless, there are modifications that can alter the biological functions in the immune system in viral infections and neoplastic diseases. The m^1^A methyltransferase TRMT61A can enhance the tumor-killing efficacy of CD8^+^ T cells through the regulation of cholesterol biosynthesis (124). Given that m^1^A modification shares several regulatory proteins with m^6^A modification, the research methodologies and findings associated with m^6^A can serve as a valuable reference to address the existing knowledge gaps in m^1^A modification within the context of autoimmune diseases. CD4^+^ T cells serve as the primary target for Human Immunodeficiency Virus (HIV) infection. During the latent phase of HIV infection, the m^5^C methyltransferase NSUN1 targets HIV TAR RNA and generates m^5^C methylation, which can impede HIV transcriptional and viral latency in CD4^+^ T cells (125). NSUN3 facilitates the infiltration of M2 macrophages while inhibiting the infiltration of M1 macrophages in head and neck squamous cell carcinoma (126). TET2 and TET3 can influence neutrophil granulation, phagocytosis, and cytokine signaling through the demethylation and destabilization of *Scos3* mRNA (127). Given the complexity of RNA modifications and the diversity of immune cells, further investigation is warranted to enhance our understanding of the interactions between RNA modifications and immune cells.

Currently, research on m^6^Am modification in autoimmune diseases remains limited. Recent studies have highlighted the critical role of m^6^Am modification in inflammatory responses (128). The deletion of the m^6^Am methyltransferase *Pcif1* mRNA impairs macrophage phagocytosis and migration via m^6^Am-CSF1R signaling, consequently mitigating periodontal inflammation (129). Although these studies are preliminary explorations, they offer valuable insights into the biological mechanisms by which m^6^Am responds to inflammation and immune cells, thereby providing a foundation for further investigation into its role in autoimmune diseases.

Despite advancements in understanding the intricate self-regulation mechanism of T cells, the significant knowledge gaps persist. Further investigations are required to determine the positions of different RNA modifications within the T cell transcriptome, identify target RNAs affected by these modifications, assess their influence on thymus cell development, and evaluate their impact on TCR signaling in mature T cells. Notably, the upstream mechanism of these enzyme activity changes should also be taken into consideration, and targeting the upstream factors of these enzymes will contribute to innovative improvements in treatment. Although these works are expected to be complex and challenging, they will hold great potential for exploring therapeutic targets.

Today, there are still many questions to be answered in order to better understand the impact of RNA modification on autoimmune diseases and T cell biology, and to apply the research to the clinic. First, investigating the extent to which RNA modifications influence disease progression in autoimmune diseases and determining whether they serve as primary regulators or auxiliary factors will be crucial for identifying potential therapeutic targets. Second, how to target RNA modification in clinical practice remains a challenge, although experiments have shown that it can work *in vivo* by giving nanoparticle preparations (130). Third, we cannot ignore the interaction between immune cells, and RNA modification may cause changes in immune function in all aspects by targeting one type of immune cell.

In cancer research, RNA enzymes are used as potential targets for therapy. For example, one preclinical study reported the inhibitory effect of FTO inhibitors on acute myeloid leukaemia (AML) cells by selectively inhibiting the m^6^A demethylase activity of FTO (131). The synthesis of selective and potent inhibitors of these enzymes is of technically feasible, it also heralds a viable new opportunity for autoimmune disease treatment. Many evidences linking dysregulation of RNA modification and autoimmune diseases suggest that developing inhibitors that target the RNA modification pathway would be fruitful. The diversity of RNA modifications and the involved molecular pathways give us hope and challenge. However, there are significant challenges in drug development, such as the selectivity of enzyme inhibitors. This problem is an inevitable challenge in the development of epigenetic modification enzyme inhibitors. For example, histone deacetylase (HDAC) is one important enzyme in the process of histone modification, which can remove the acetyl group on the histone Lysine residue, change the electric charge to make the chromosome structure more tightly, and thus inhibit the transcriptional expression of genes (132). To date, a total of five HDAC inhibitors have received market approval globally. Among these, only one is a selective inhibitor, while the remaining four are pan-inhibitors of HDAC. HDAC drugs have made a breakthrough in the clinical treatment of various subtypes of blood tumors, but the treatment of non-blood solid tumors is not effective (133). With the in-depth study of RNA modification, we have not only gained a deeper understanding of the disease mechanism and its target pathways but also uncovered its intricate relationship with the occurrence and immunity of autoimmune diseases. Many challenges remain due to the complexity of post-transcriptional modifications, epigenetic transcriptional regulation, and non-epigenetic cell signaling cascades. It is believed that with the continuous efforts of scientists, more and more efficient and low-toxic enzyme inhibitors will be applied to the treatment of autoimmune diseases and other diseases.
