# Supplementary material for: RNA modification: a promising code to unravel the puzzle of autoimmune diseases and CD4+ T cell differentiation
Source: Front Immunol. 2025 Mar 24;16:1563150. doi: 10.3389/fimmu.2025.1563150 (PMC11973318; doi:10.3389/fimmu.2025.1563150)
Supplement: Supplementary file 1 [file Table1.docx]

**Supplementary Table 1. An overview of the general characteristics of major types of RNA modifications**

| **Type** | **Class** | **Protein** | **Targets** | **Function** | **Reference** |
| --- | --- | --- | --- | --- | --- |
| m^6^A | writer | METTL3 | mRNA, rRNA, snRNA, miRNA, IncRNA, circRNA | Catalyzes the majority of m^6^A modifications by forming a methyltransferase complex with METTL14. | ([Li et al., 2024a](#_ENREF_50)) |
|  |  | METTL14 |  | Provides critical structural support within the methyltransferase complex framework | ([Zhang et al., 2023](#_ENREF_129)) |
|  |  | WTAP |  | facilitates m^6^A modification by recruiting co-localized METTL3-METTL14 | ([Zhou et al., 2022](#_ENREF_130)) |
|  |  | RBM15/15B |  | Binds the m6A complex and recruit it to a specific RNA site |  |
|  |  | ZC3H13 |  | Promotes methyltransferase complex RNA binding |  |
|  |  | VIRMA |  | Provides binding sites for METTL3/METTL14 |  |
| m^6^Am  m^1^A | eraser  reader  write  eraser  write | METTL16  FTO  ALKBH5  YTHDF1  YTHDF2  YTHDF3  YTHDC1  YTHDC2  IGF2BP1/2/3  HNRNPs  PCIF1  METTL4  FTO  TRMT61B  TRMT10C  TRMT6/61A  TRMT61B | mRNA snRNA  tRNA, rRNA, lncRNA, mRNA | Catalytic m^6^A of snRNA and other lncRNA  Removes m^6^A/m^6^Am/m^1^A modifications  Specifically demethylates the m^6^A modification  Stabilizes transcripts and initiates the translation process  Facilitates the process of degradation  Facilitates the processes of translation and degradation  Regulate RNA splicing, nuclear export, and degradation.  Enhances translation efficiency and degradation  Stabilizes RNA transcripts and enhances translational efficiency  Regulate splicing  Facilitates m^6^Am  Facilitates m^6^Am  Demethylate m^6^A/m^6^Am/m^1^A modifications  Facilitates mA at m^1^A58  Facilitates mA at m^1^A9  Facilitates m^1^A  Facilitates m^1^A | ([van Vroonhoven et al., 2023](#_ENREF_99))  ([Teng et al., 2023](#_ENREF_95))  ([Benak et al., 2023](#_ENREF_5))  ([Li et al., 2022](#_ENREF_49))  ([Barraud et al., 2008](#_ENREF_3)) |

**Supplementary Table 1. (Continue)**

| **Type** | **Class** | **Protein** | **Targets** | **Function** | **Reference** |
| --- | --- | --- | --- | --- | --- |
|  | eraser | ALKBH1 |  | Catalyzes the demethylation of the majority of m^1^A residues in cytoplasmic tRNAs. | ([Sharma et al., 2018](#_ENREF_86)) |
|  |  | ALKBH3 |  | Catalyzes the demethylation of the m^1^A residues in tRNA and mRNA |  |
|  |  | ALKBH7 |  | Catalyzes the demethylation of the m^1^A within the mitochondrial Leut pre-tRNA regions |  |
|  |  | FTO |  | Demethylates m^6^A/m^6^Am/m^1^A modifications |  |
|  | reader | YTHDF1/2/3 |  | Regulates RNA splicing, nuclear export, and degradation. |  |
|  |  | YTHDC1 |  | Regulates RNA splicing, nuclear export, and degradation. |  |
| m^5^C | write | NSUN1 | tRNA, rRNA, ncRNA, lncRNA, mRNA, mt-tRNA, viral-RNA,  mRNA, tRNA, rRNA, miRNA  tRNA, rRNA, snRNA | Facilitates m^5^C in rRNA | ([Nombela et al., 2021](#_ENREF_74)) |
| m^7^G  Nm | eraser  reader  write  write | NSUN2  NSUN3  NSUN4  NSUN5  NSUN6  DNMT2  ALKBH1  TET1/2/3  ALYREF  YTHDF2  METTL1-WDR4 complex  RNMT-RAM complex  FTSJ1 |  | Facilitates the majority of m^5^C residues  Facilitates m^5^C in mt-tRNA  Facilitates m^5^C in rRNA  Facilitates m^5^C in rRNA  Facilitates m^5^C in tRNA  Facilitates m^5^C in tRNA  Demethylates the m^5^C  Catalyzes first step of m^5^C  Facilitates the nuclear export process of RNA  Regulates the maturation process  Catalyzes m^7^G  Facilitates Nm | ([Guallar et al., 2018](#_ENREF_29))  ([Yang et al., 2017](#_ENREF_122))  (  [Cai et al., 2023](#_ENREF_7))  ([Chen et al., 2022b](#_ENREF_14))  ([Brazane et al., 2023](#_ENREF_6)) |

m^6^A: N6-methyladenosine, m^6^Am: N6-2'-O-dimethyladenosine, m^1^A: N1-methyladenosine, m^5^C: 5-methylcytidine, m^7^G: N7-methylguanosine, Nm: 2'-O-methylation
