# Supplementary material for: RNA modification: a promising code to unravel the puzzle of autoimmune diseases and CD4+ T cell differentiation
Source: Front Immunol. 2025 Mar 24;16:1563150. doi: 10.3389/fimmu.2025.1563150 (PMC11973318; doi:10.3389/fimmu.2025.1563150)
Supplement: Supplementary file 2 [file Table2.docx]

**Supplementary Table 2. Changes of RNA modification related enzymes in autoimmune diseases.**

| **Disease** | **Sample** | **Changes compared with HCs** | **Target gene or signaling pathway** | **References** |
| --- | --- | --- | --- | --- |
| SLE | Peripheral blood | METTL3*↓* METTL14*-* WTAP*↓* FTO↓ ALKBH5*↓* YTHDF2*↓* | unclear | ([Luo et al., 2020a](#_ENREF_56)) |
|  | PBMCs | METTL3*-* METTL14↓ WTAP- FTO- ALKBH5*↓* YTHDF2*↓* | unclear | ([Luo et al., 2020c](#_ENREF_59)) |
| RA | Peripheral blood | METTL14↑ | MAPK signaling pathway | ([Zhu and Wan, 2024](#_ENREF_123)) |
|  |  | METTL3- METTL14- WTAP- FTO↓ALKBH5↓YTHDF2↓ | unclear | ([Luo et al., 2020b](#_ENREF_57)) |
|  | PBMCs | METTL14↓ | *TNFAIP3* | ([Tang et al., 2023](#_ENREF_84)) |
|  |  | *YTHDF2*↓ | *NLRP3* | ([Xiao et al., 2023](#_ENREF_104)) |
|  |  | METTL3↑IGF2BP3↑ | unclear | ([Wang et al., 2019b](#_ENREF_93), [Fan et al., 2022](#_ENREF_21)) |
|  | Synovial tissues | METTL3↑ | NF-κB signaling  pathway | ([Shi et al., 2021](#_ENREF_79)) |
|  |  | METTL14↑ WTAP↑ FTO↑ ALKBH5↑ IGFBP3↑ IGFBP2↑ | unclear | ([Su et al., 2024](#_ENREF_82), [Miao et al., 2024](#_ENREF_65), [Shi et al., 2021](#_ENREF_79), [Fan et al., 2022](#_ENREF_21), [Xiao et al., 2022a](#_ENREF_103), [Zhu and Wan, 2024](#_ENREF_123)) |
|  | FLSs | METTL3↑  FTO↑  ALKBH5↑ | *AMIGO2*  *ADAMTS15*  *JARID2* | ([Miao et al., 2024](#_ENREF_65)) |
|  |  | METTL14↑ WTAP↑ | unclear | ([Su et al., 2024](#_ENREF_82), [Miao et al., 2024](#_ENREF_65), [Li et al., 2023b](#_ENREF_47), [Liu et al., 2024](#_ENREF_49), [Li et al., 2024b](#_ENREF_45), [Kuang et al., 2024](#_ENREF_38)) |
|  | macrophages | METTL3↑ METTL14↑ | NF-κB signaling pathway | ([Wang et al., 2019b](#_ENREF_93)) |
|  |  | METTL14↑ | unclear | ([Wang et al., 2019b](#_ENREF_93), [Miao et al., 2024](#_ENREF_65), [Zhu and Wan, 2024](#_ENREF_123)) |

**Supplementary Table 2. (Continue)**

| **Disease** | **Sample** | **Compared with HCs** | **Target gene or signaling pathway** | **References** |
| --- | --- | --- | --- | --- |
| IBD | Pathological tissue | METTL3↑ METTL14↑ | NF-κB signaling pathway | ([Yang et al., 2022](#_ENREF_110), [Zhang et al., 2022](#_ENREF_118)) |
|  |  | FTO↓ | *Cers6* | ([Ma et al., 2024](#_ENREF_64)) |
|  |  | WTAP↑ IGF2BP1*↓* IGF2BP2*↓* | unclear | ([Ge et al., 2024](#_ENREF_24), [Chen et al., 2021](#_ENREF_12)) |
|  | macrophages | METTL3↑ | *PGP* | ([Yin et al., 2024a](#_ENREF_116)) |
| AS | PBMCs | WTAP↑ ALKBH5↓ YTHDF2↓ HNRNP↓ | unclear | ([Wu et al., 2024](#_ENREF_102), [Luo et al., 2022](#_ENREF_58)) |
|  | T cell | METTL14↓ | *FOXO3a* | ([Chen et al., 2023](#_ENREF_14)) |
| pSS | Peripheral blood | ALKBH5↓ RBMX↓ RBM15B↓ YTHDF2↓ | unclear | ([Cheng et al., 2022](#_ENREF_15)) |
|  | PBMCs | METTL3↑ ALKBH5↑ | unclear | ([Ma et al., 2023](#_ENREF_62), [Yin et al., 2024b](#_ENREF_117), [Xiao et al., 2022c](#_ENREF_106)) |
|  | T cells | METTL3↑ ALKBH5↑ YTHDF2↑ |  | ([Yin et al., 2024b](#_ENREF_117)) |
|  | salivary gland epithelial cells | METTL3↑ METTL14↑ |  | ([Truffinet et al., 2024](#_ENREF_87)) |

↑: up-regulated ↓: down-regulated *-*:with no statistical difference HCs: healthy controls SLE: systemic lupus erythematosus PBMCs: peripheral blood mononuclear cells RA: rheumatoid arthritis FLSs: fibroblast-like synovial cells IBD: inflammatory bowel disease AS: ankylosing spondylitis pSS: primary Sjogren's syndrome
